# Web-Based Aftercare for Women With Bulimia Nervosa Following Inpatient Treatment: Randomized Controlled Efficacy Trial

**DOI:** 10.2196/jmir.7668

**Published:** 2017-09-22

**Authors:** Corinna Jacobi, Ina Beintner, Eike Fittig, Mickey Trockel, Karsten Braks, Carmen Schade-Brittinger, Astrid Dempfle

**Affiliations:** ^1^ Institut für Klinische Psychologie und Psychotherapie Professur Klinische Psychologie & E-Mental Health Technische Universität Dresden Dresden Germany; ^2^ Celenus Klinik Carolabad Medizinisches Rehabilitationszentrum für Psychotherapie, Psychiatrie und Psychosomatik Chemnitz Germany; ^3^ Department of Psychiatry and Behavioral Sciences Stanford University School of Medicine Stanford, CA United States; ^4^ Klinik am Korso gGmbH Bad Oeynhausen Germany; ^5^ Koordinierungszentrum für Klinische Studien Marburg Philipps-Universität Marburg Marburg Germany; ^6^ Institut für Medizinische Informatik und Statistik Universitätsklinikum Schleswig-Holstein Christian-Albrechts-Universität zu Kiel Kiel Germany

**Keywords:** bulimia nervosa, web-based intervention, aftercare, relapse

## Abstract

**Background:**

Relapse rates in bulimia nervosa (BN) are high even after successful treatment, but patients often hesitate to take up further treatment. An easily accessible program might help maintain treatment gains. Encouraged by the effects of Web-based eating disorder prevention programs, we developed a manualized, Web-based aftercare program (IN@) for women with BN following inpatient treatment.

**Objective:**

The objective of this study was to determine the efficacy of the web-based guided, 9-month, cognitive-behavioral aftercare program IN@ for women with BN following inpatient treatment.

**Methods:**

We conducted a randomized controlled efficacy trial in 253 women with DSM-IV (Diagnostic and Statistical Manual of Mental Disorders, fourth edition) BN and compared the results of IN@ with treatment as usual (TAU). Assessments were carried out at hospital admission (T0), hospital discharge/baseline (T1), postintervention (T2; 9 months after baseline), 9-month follow-up (T3; 18 months after baseline). The primary outcome, abstinence from binge eating and compensatory behaviors during the 2 months preceding T2, was analyzed by intention to treat, using logistic regression analyses. Frequencies of binge eating and vomiting episodes, and episodes of all compensatory behaviors were analyzed using mixed effects models.

**Results:**

At T2, data from 167 women were available. There were no significant differences in abstinence rates between the TAU group (n=24, 18.9%) and the IN@ group (n=27, 21.4%; odds ratio, OR=1.29; *P*=.44). The frequency of vomiting episodes in the IN@ group was significantly (46%) lower than in the TAU group (*P*=.003). Moderator analyses revealed that both at T2 and T3, women of the intervention group who still reported binge eating and compensatory behaviors after inpatient treatment benefited from IN@, whereas women who were already abstinent after the inpatient treatment did not (*P*=.004; *P*=.002). Additional treatment utilization was high in both groups between baseline and follow-up.

**Conclusions:**

Overall, data from this study suggest moderate effects of IN@. High rates of outpatient treatment utilization after inpatient treatment may have obscured potential intervention effects on abstinence. An aftercare intervention might be more beneficial as part of a stepped-care approach.

**Trial Registration:**

International Standard Randomized Controlled Trial Number (ISRCTN): 08870215; http://www.isrctn.com/ISRCTN08870215 (Archived by WebCite at http://www.webcitation.org/6soA5bIit)

## Introduction

Bulimia nervosa (BN) is a severe disorder associated with serious medical morbidity and psychosocial comorbidity [1,2]. For a substantial proportion of patients, the long-term course is chronic; depending on outcome criteria and follow-up duration, less than half of the patients achieve full recovery [3,4]. Several studies indicate that higher frequencies of binge eating and purging at baseline, postintervention, and follow-up are negative prognostic factors [5-11].

In the past decades, a number of effective psychological treatments for BN have been developed and are available [12]. Both US (APA) and European guidelines [12,13] recommend cognitive behavioral therapy (CBT [14]) as first-line treatment. Meta-analytic reviews [13,15,16] show good effects for eating disorder attitudes and moderate remission rates ranging between 20% and 30% for CBT. However, relapse rates within the first months of treatment are high even after significant reduction of core BN symptoms [8,17-19], and some women may even be reluctant to seek further treatment after experiencing relapses [20]. Maintaining treatment gains remains a challenge especially for the more severe and chronically ill BN patients [21].

Outside the eating disorders field, technology-enhanced approaches (ie, interventions delivered through the Internet or mobile apps) have been increasingly utilized as aftercare interventions following in- or outpatient treatment across mental health disorders (eg, [22-25]) or for specific disorders such as bipolar disorder [26] or pain disorder [27].

Compared with face-to-face interventions, Web-based interventions offer several potential advantages [28]. They can overcome existing barriers such as cost, service availability, wait time, transportation, and stigma, thus countering health care–related disparities, and can be easily tailored to individual needs. Greater anonymity may encourage individuals to seek help and reveal more sensitive health information. Finally, Web-based programs might be more cost-effective compared with face-to-face interventions [29] and have high user acceptability.

In the field of indicated prevention of eating disorders, Web-based approaches have proven to be efficacious in reducing core symptoms of eating disorders, including high weight and shape concerns, body dissatisfaction [30,31], binge eating [32], new onset of full-syndrome eating disorders [33,34], and shown promise for improving low body weight and restrained eating [35]. However, at the time of initiation of the current trial, no technology-based intervention was available for the maintenance of in- or outpatient treatment gains and prevention of relapses, and in the meantime, very few randomized controlled trials have utilized and tested the efficacy of technology-enhanced interventions following in- or outpatient treatment.

In an aftercare program based on text messaging [36], 165 German patients with BN were randomized to an intervention group (16-week short message service (SMS)–based maintenance) or a treatment as usual (TAU) control group following inpatient treatment. Remission rates at 8 months after hospital discharge were significantly higher in the intervention group than in the TAU group, independent of utilization of outpatient treatment. For patients of the intervention group who did not use any additional outpatient treatment, remission rates were even higher. Another German study [37] examined the efficacy of a Web-based relapse prevention program (RP) over 9 months after inpatient treatment in 258 women with anorexia nervosa (AN), randomized to the RP or TAU condition. At postintervention, RP completers had gained significantly more body weight (0.62 BMI points) than patients in the TAU condition and showed significant improvements in specific eating-related cognitions and behaviors. At 9-month follow-up, the subgroup of participants with high adherence to the program (“full completers”) achieved further improvements in body mass index (BMI) [38]. Finally, following an earlier pilot study with promising results conducted in Hungary, the effects of a Web-based support program following routine outpatient and inpatient care were also evaluated in a recent larger, randomized controlled trial [39]. A total of 105 women with BN and eating disorders not otherwise specified were randomly assigned to an immediate Web-based support program over 4 months or to a 4-month waiting-list TAU control condition. Both groups showed significant reductions in eating disorder-related attitudes at postintervention compared with baseline, with no significant effects of intervention on improvement.

Overall, the results of studies directed at the maintenance of treatment gains for different ED symptoms using technology-based interventions are encouraging, but do not show clear superiority for a relevant outcome measure. These trials also demonstrated that a substantial proportion of patients (28-93%) had utilized additional treatment [36,37,39].

Encouraged by the high acceptance [40] and efficacy of previously evaluated, Web-based indicated prevention programs for eating disorders [30,32,35], we decided to develop an Web-based aftercare program (IN@) for women with BN following inpatient treatment [41]. The aim of this study was to evaluate the efficacy of this program in maintaining or achieving abstinence from core BN symptoms in comparison with a TAU control group. We hypothesized that the aftercare intervention would lead to larger and more stable effects in core BN symptoms compared with TAU.

## Methods

### Study Design and Participants

We conducted a randomized, controlled efficacy trial in women with DSM-IV BN. The trial protocol, summarizing details on study design and intervention, has been published elsewhere [41]. Over a 4½-year period, patients were screened and recruited from 13 psychosomatic hospitals offering specialized inpatient treatment for eating disorders throughout Germany. Patients were eligible for inclusion if they were aged 17 years or older, fulfilled DSM-IV-TR criteria for BN at hospital admission, and had successfully completed inpatient treatment, defined by a reduction of binge eating and compensatory behaviors by at least 50% based on the past 2 weeks before discharge compared with admission. Patients were excluded if their BMI had dropped below 17.5 kg/m^2^ during the inpatient treatment or if local hospital staff regarded them as unfit to participate in a Web-based program (eg, due to psychotic symptoms, acute suicidality, severe personality disorders, or language barriers).

Assessment points were as follows: prebaseline (T0; hospital admission), baseline (T1; hospital discharge), postintervention (T2; 9 months after baseline), and 9-month follow-up (T3; 18 months after baseline). Patients received up to 80€ for participating in all assessments, whereas there were no monetary incentives for using the Web-based intervention.

The study was approved by the local ethics committee of TU Dresden and by ethics committees of all other federal states in which participating hospitals were located. Written informed consent was obtained from all patients and—in case of minors—their legal guardians. The study was conducted according to the Declaration of Helsinki and Good Clinical Practice principles.

Quality-control methods comprised case-report forms, independent data management, on-site monitoring, and documentation of adverse and severe adverse events. Data management was provided by the independent Clinical Trials Center in Marburg and included regular checks for consistency and plausibility, and queries if inconsistencies or missing data became evident.

### Randomization and Masking

Concealed randomization was carried out centrally by the independent Clinical Trials Center in Marburg after patients had been enrolled in the study and had given informed consent in a ratio of 1:1. The randomization was done with permuted blocks stratified by Center. Patients and psychologists involved in the moderation of the aftercare program could not be masked to intervention allocation. Assessors who carried out T0-T3 diagnostic assessments were blind to intervention allocation and neither involved in the moderation of the intervention nor in the final data analyses.

### Procedures

Within the first 2 weeks after hospital admission, potentially eligible patients were contacted by hospital staff and informed about the study. Patients willing to participate were interviewed over the phone by trained interviewers at TU Dresden to confirm a DSM-IV diagnosis of BN and to assess comorbidity and received login data to access the password protected Web-based trial platform. During the week before or after discharge, patients were interviewed and asked to complete the Web-based assessments again. Patients who had reduced their core BN symptoms by 50% compared with hospital admission were then randomized to the IN@ or TAU condition.

### The Intervention IN@

We designed IN@ to target the maintenance of inpatient treatment gains and reduce relapses after discharge. The intervention was based on principles of cognitive behavioral treatment for BN [14] and covered topics such as, eating behaviors and core bulimic symptoms, healthy exercise, body image, self-esteem, emotional and social skills. Table 1 provides an overview of the program content. Additional interactive features of the program were a monitoring log for bulimic symptoms, a body image, and a personal diary. Three clinical psychologists trained in behavior therapy for eating disorders guided the program, that is, provided individualized email feedback to entries in diaries and offered up to 9 monthly real-time individual chats of approximately 1 hour per participant. The program consisted of 11 Web-based sessions over 9 months with fortnightly sessions scheduled during the first 2 months after hospital discharge and monthly sessions thereafter.

The program’s home page also provided an overview of all participating hospitals and CVs of the program moderators once patients had logged in to facilitate the credibility of the intervention. Screenshots of the intervention are provided in Multimedia Appendices 1 and 2.

### Treatment as Usual (TAU)

Patients assigned to the TAU group were assessed at all assessment points but did not receive additional treatment recommendations from the research team. However, most hospitals recommend some form of outpatient treatment following inpatient treatment. For ethical reasons, we did not interfere with these recommendations and subsequent decisions on concomitant treatment but documented it at all assessment points for both treatment arms.

**Table 1:** Overview of the program content.

Session	Module	Examples of content
1	Introduction	
	Eating behaviors	Personal eating disorder history
		Dietary restraint and binge eating
		Set-point theory
		Forbidden foods
2	Eating behaviors	Behavioral chain model for identifying high-risk situations
	Body image	Cultural beauty ideals and their impact on self-esteem
	Emotion regulation	Automatic thoughts and their consequences
3	Interpersonal relationships and social skills	Introduction to social skills training
	Emotion regulation	Role of emotions in driving functional and dysfunctional behaviors
4	Interpersonal relationships and social skills	Functional and dysfunctional beliefs and their impact on interpersonal relationships
	Emotion regulation	Role of emotions in triggering dysfunctional eating behaviors
	Eating behaviors	Helpful and unhelpful eating habits Dieting
5	Emotion regulation	Coping with unpleasant emotions
	Alternative behaviors	Increasing pleasant activities
	Body image	Components of body image
		Irrational beliefs regarding body image
6	Interim self-assessment	
7	Emotion regulation	Coping with stress
	Perfectionism	Irrational perfectionist beliefs
8	Body image	Avoidance behavior
	Interpersonal relationships and social skills	Dealing with critical comments
9	Emotion regulation	Mindfulness
	Body image	Mirror confrontation
	Exercise	Reduction of compulsive exercise
10	Self-assessment	
11	Summary	

### Outcome Measures

The primary outcome was defined as abstinence from any core BN symptoms (binge eating, vomiting, laxative abuse, abuse of diuretics or other medication to control weight, and driven exercise) in the past 2 months before postintervention (T2) and not having resumed inpatient treatment after T1. Information on the primary outcome was derived both from the Structured Interview for Anorexia and Bulimia nervosa (SIAB-EX) and the weekly symptom checklists. If there was contradictory information from these two sources, the patient was classified as nonabstinent. Secondary outcomes were abstinence from any core BN symptoms in the 2 months before follow-up (T3) as defined earlier, the frequencies of episodes of binge eating, vomiting, and all compensatory behaviors per week in the past 3 months before postintervention (T2) and follow-up (T3), and recovery defined as not fulfilling DSM-IV-TR diagnostic criteria for an eating disorder anymore at postintervention (T2) and follow-up (T3). As abstinence rates are strongly influenced by definitions of abstinence [17], we chose to align our definition of abstinence with previous recommendations [17]. Regarding the frequencies of binge eating and compensatory behaviors as well as recovery, we aligned our definitions with the DSM-IV-TR criteria.

Because hospitals were located nationwide and patients lived in and returned to different parts of Germany, all interview assessments were carried out over the telephone. At all assessment points, trained assessors blinded to patients’ group assignment assessed eating disorder-specific and general psychopathology. We applied the SIAB-EX [42] to measure eating disorder symptomatology and general psychopathology and ascertain the diagnosis of BN at all assessment points. The interview was slightly modified by adding continuous items measuring the absolute frequencies (rather than categorical items of frequency spans) of binge eating and compensatory behaviors over periods of 2 weeks, 2 months, and 3 months. At prebaseline, we also used the Structured Clinical Interview for DSM-IV axis I mental disorders (SCID-I [43]) to measure psychiatric comorbidity. All interview documentations were monitored and checked for plausibility by the study coordinator and the independent monitor, and assessors received regular feedback based on this monitoring.

Questionnaire assessments (for further moderator as well as mediator analyses) and the weekly symptom checklists were integrated into the Web-based platform that also hosted the intervention. Patients in both the intervention and the TAU group were prompted regularly to report the frequencies of all core BN symptoms.

At T2 and T3, we also assessed the amount of any additional in- and outpatient treatment patients had utilized during the IN@ intervention and the follow-up period (in-, out-, day-patient therapy, days of treatment, number of outpatient treatment sessions, and number of therapists).

### Statistical Analyses

Sample size calculations were based on average rates of patients who do not remain abstinent of binge eating and compensatory behaviors after having achieved abstinence during treatment [17]. We assumed an average abstinence rate of 15% for TAU and of 35% for IN@, that is, we considered a difference of 20 percent points between groups, a clinically relevant reduction in core BN symptomatology. Using standard sample size formulae for the comparison of two rates by Fisher exact test at an alpha-level of 5% (two-sided) and statistical power of 85%, we calculated the minimum sample size to be n=90 for each group or a total of N=180 patients. Assuming a dropout rate of 30% at postintervention, a total of N=258 patients had to be included in the study.

We analyzed the primary outcome by intention to treat (ITT), which included all patients who had been randomized, using logistic regression analyses adjusted for prebaseline binge eating frequency, frequency of compensatory behaviors, BMI, SIAB general psychopathology, and baseline abstinence. If no data (from SIAB-EX or weekly symptom checklist) were available for a patient or if data from both sources were contradictory, she was considered nonabstinent (worst case imputation). The primary outcome was also analyzed using the complete cases (CCs) that included only women who had completed postintervention assessments and the per-protocol sample (PP) which included all women of the IN@ group who had completed postintervention interview assessments and had actively engaged with the intervention, that is, opened at least 25% of program pages or participated in at least two one-to-one chats. For the TAU group, the PP was identical with the CC. Abstinence at follow-up was analyzed in the same manner. Group differences were analyzed by logistic regression analyses, adjusting for prebaseline binge eating frequency, frequency of compensatory behaviors, BMI and SIAB general psychopathology, and baseline abstinence.

We analyzed the frequencies of episodes of binge eating, vomiting, and of all compensatory behaviors per week in the past 3 months preceding baseline (T1, discharge), postintervention (T2), and follow-up (T3) using mixed effects model to account for the nested data structure of three observations across time within individual participants [44]. Longitudinal mixed effects modeling is the method of choice for intent-to-treat analyses to assess clinical trial outcomes [45]. Multiple imputation before conducting longitudinal mixed effects modeling does not add value and is not necessary [46,47]. Episodes of binge eating, vomiting, and other compensatory behaviors were measured as count data and modeled as log-linked dependent variables to estimate intervention effects on change in rate from baseline to T2 or T3.

The percentage of women who did not fulfill diagnostic criteria for any eating disorders at T2 and T3 were compared between the groups by Fisher exact test.

Moderator analyses were performed for abstinence (from binge eating and compensatory behaviors) at postintervention and follow-up using mixed effects model. Because IN@ represents a novel aftercare approach and findings on patient characteristics as predictors of outcome for ED treatment are inconclusive, we decided to employ an exploratory approach as proposed by the MacArthur Foundation [48,49]. Accordingly, in a first step, we calculated correlations (Kendall’s Tau) between all available prebaseline and baseline variables and abstinence at postintervention and follow-up. A variable was considered a potential moderator if the correlation between the variable and abstinence at postintervention or follow-up was >.20. In a second step, each candidate moderator variable was entered separately into a mixed effects model. A variable was considered a moderator if there was a significant group × variable interaction effect on either postintervention or follow-up abstinence. Finally, if abstinence was moderated by more than one variable, all moderators were entered in a mixed effects analysis to determine the final prediction model. Statistical analyses were performed using SPSS 22 (IBM, Armonk, NY) and HLM7 (Scientific Software International, Inc., Skokie, IL).

## Results

### Patient Flow and Characteristics

**Figure 1:**
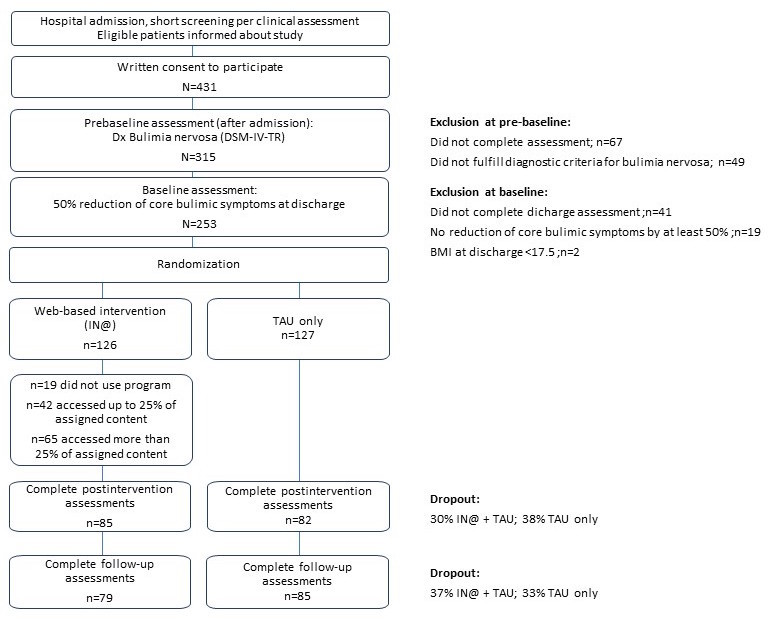
Study design and recruitment.

Between 2007 and 2012, about 2500 patients were admitted for eating disorder treatment to collaborating hospitals and assessed for eligibility. A total of 431 women gave written informed consent to participate in the trial, of which 364 were reached for the prebaseline assessments, and of those, 315 fulfilled DSM-IV-TR criteria for BN (Figure 1).

Inpatient treatment comprised CBT-oriented (22%) and mixed (psychodynamic with CBT-elements; 78%) treatment approaches. At T1, 274 patients completed the baseline assessment and of those, 253 patients had reduced their core BN symptoms by at least 50% and were thus included in the trial (which would still result in 84% power under the initial planning assumptions); 126 were randomized to IN@ and 127 to TAU. At T2, 88 patients of the intervention group and 79 patients of the control group had completed postintervention assessments resulting in a dropout rate of 30% in the IN@ group and 38% in TAU. At T3, 82 patients of the IN@ group and 81 of the TAU group had completed follow-up assessments resulting in an overall dropout rate of 34.9% in IN@ and 36.2% in TAU.

Table 2 summarizes baseline characteristics of all patients. There were no relevant baseline differences between groups regarding BMI, age, illness duration, frequency of core BN symptoms, and frequency of any prior (in- and outpatient) psychiatric or psychotherapeutic treatment. However, a history of AN was more prevalent in the TAU group (45.6% vs 32.5%), whereas a current anxiety disorder was more prevalent in the IN@ group (46.0% vs 32.3%) and a current substance use disorder was more prevalent in the TAU group (6% vs 1%). Approximately 85.3% of patients had already received some kind of psychiatric or psychotherapeutic treatment before hospital admission.

Overall, patients’ BMI was in the normal range. Patients reported illness duration of 7.2 years on average and reported high levels of binge eating and vomiting episodes. In the 2 weeks preceding hospital discharge (baseline), the average number of objective binge eating and vomiting episodes per week had dropped considerably and 54.9% of patients were abstinent from binge eating and compensatory behaviors. Current or lifetime comorbidity was high.

### Program Adherence

In total, 19 (15.1%) intervention participants never logged on to the program. Of the remaining 107 women, 46 (36.5%) accessed at least half of the intervention content. On average, participants opened 36% of the program pages (Median: 28%) and accessed 5 of 11 sessions (Median: 4 sessions). Furthermore, 47 women (37.3%) took part in at least one live chat. More detailed information on adherence and therapist time invested will be reported separately.

### Effects of the Intervention on Abstinence Rates and Core BN Symptoms

The analysis of the primary outcome showed that at postintervention, about 1 in 5 patients in the ITT sample reported abstinence from binge eating and any compensatory behaviors during the previous 2 months, with no significant difference between the intervention group (21.4%) and the TAU group (18.9%; Table 3). Additional analyses also revealed no significant differences between the groups: In the CC sample (n=167), abstinence rates were 31.0% in the intervention group and 28.2% in the TAU group (*P*=.53). In the PP sample (n=68), the abstinence rate was 30.1% (*P*=.76). Abstinence rates at follow-up (a secondary outcome) remained stable, with no significant difference between the intervention group (22.2%) and the TAU group (17.3%).

**Table 2:** Sample key characteristics at hospital admission by group.

Patient characteristics	IN@^a^, n=126	TAU^b^, n=127
Body mass index, in kg/m^2^, at prebaseline; mean (SD)^c^	21.49 (2.96)	21.99 (3.85)
Age, in years, at prebaseline; mean (SD)^c^	25.67 (7.18)	26.26 (6.92)
Duration of illness in years at prebaseline, mean (SD)^c^	6.62 (5.59)	7.65 (6.28)
Objective binge eating episodes per week in the past 3 months at prebaseline, mean (SD)^c^	13.72 (11.93)	15.67 (17.11)
Objective binge eating episodes per week in the past 2 weeks at baseline, mean (SD)^c^	0.48 (1.37)	0.66 (2.09)
Vomiting episodes per week in the past 3 months at prebaseline, mean (SD)^c^	18.10 (19.67)	18.73 (20.44)
Vomiting episodes per week in the past 2 weeks at baseline, mean (SD)^c^	0.63 (1.47)	0.80 (2.16)
Episodes of all compensatory behaviors per week in the past 3 months at prebaseline, mean (SD)^c^	22.57 (20.31)	23.39 (20.13)
Episodes of all compensatory behaviors per week in the past 2 weeks at baseline, mean (SD)^c^	1.49 (2.48)	1.71 (2.96)
Abstinent from binge eating and compensatory behaviors in the past 2 weeks at baseline, n (%)^c^	68 (54.0)	71 (55.9)
Any prior psychotherapeutic or psychiatric treatment, n (%)	107 (84.9)	107 (84.2)
History of anorexia nervosa (AN), n (%)^c^	41 (32.5)	58 (45.6)
Current affective disorder at prebaseline, n (%)^d^	50 (39.7)	44 (34.6)
Current anxiety disorder at prebaseline, n (%)^d^	58 (46.0)	41 (32.3)
Current substance abuse disorder at prebaseline, n (%)^d^	1 (0.7)	7 (5.5)

^a^IN@: Web-based aftercare intervention.

^b^TAU: Treatment as usual.

^c^According to modified Structured Interview for Anorexia and Bulimia nervosa (SIAB-EX [42]).

^d^According to Structured Clinical Interview for DSM-IV axis (SCID [43]).

**Table 3:** Abstinence rates by group at postintervention and follow-up (intent-to-treat analysis).

Outcome of abstinence from binge eating and all compensatory behaviors in the past 2 months^a,e^	IN@^b^, n=126 n (%)	TAU^c^, n=127 n (%)	Odds ratio (95% CI)^d^	*P* value
At postintervention^f^	27 (21.4)	24 (18.9)	1.29 (0.68-2.44)	.44
At follow-up^f^	28 (22.2)	22 (17.3)	1.49 (0.77-2.86)	.24

^a^Patients who did not provide data were classified as nonabstinent.

^b^IN@: Web-based aftercare intervention.

^c^TAU: Treatment as usual.

^d^Abstinence was coded as 1, nonabstinence as 0 in the model.

^e^No binge eating and compensatory episodes in the past 2 months according to modified structured interview for anorexia and bulimia nervosa (SIAB-EX [42]) and weekly symptom checklist.

^f^Group effect in logistic regression analysis adjusted for prebaseline binge eating frequency, frequency of compensatory behaviors, body mass index (BMI), and SIAB general psychopathology and baseline abstinence.

**Table 4:** Frequencies of binge eating, vomiting, and all compensatory behaviors: final estimation of fixed effects (population-average model with robust standard errors).

Fixed effect				Coefficient		Standard error		*t* ratio		Event rate ratio (95% CI )		*P* value
**Frequency of objective binge eating episodes**												
	Intercept			−0.56		0.194		−2.872		0.57 (0.390-0.843)		.005
	**Postintervention slope**											
		Intercept	2.10		0.214		9.800		8.16 (5.353-12.450)		<.001	
		IN@ intervention effect	−0.32		0.178		−1.810		0.72 (0.510-1.029)		.07	
	**Follow-up slope**											
		Intercept	1.98		0.222		8.909		7.23 (4.673-11.216)		<.001	
		IN@ intervention effect	0.03		0.670		0.040		1.03 (0.274-3.851)		.97	
**Frequency of vomiting episodes**												
	Intercept			−0.33		0.161		−2.043		0.72 (0.522-0.992)		.04
	**Postintervention slope**											
		Intercept	2.39		0.216		11.070		10.94 (7.147-16.746)		<.001	
		IN@ intervention effect	−0.61		0.208		−2.951		0.54 (0.359-0.815)		.003	
	**Follow-up slope**											
		Intercept	2.49		0.241		10.297		12.03 (7.479-19.378)		<.001	
		IN@ intervention effect	−0.53		0.407		−1.292		0.59 (0.265-1.318)		.20	
**Frequency of episodes of all compensatory behaviors**												
	Intercept			0.47		0.107		4.399		1.60 (1.294-1.981)		<.001
	**Postintervention slope**											
		Intercept	1.79		0.176		10.167		6.01 (4.246-8.508)		<.001	
		IN@ intervention effect	−0.36		0.187		−1.910		0.70 (0.483-1.011)		.06	
	**Follow-up slope**											
		Intercept	1.94		0.184		10.565		6.96 (4.847-9.993)		<.001	
		IN@ intervention effect	−0.44		0.292		−1.490		0.65 (0.364-1.151)		.14	

The frequency of binge eating episodes (Table 4) increased significantly in both groups after hospital discharge. At postintervention, the frequency of binge eating episodes in the intervention group was 28% lower than in the TAU group (3.4 vs 4.7 episodes per week), but the difference was not statistically significant. At follow-up, there was no difference between the groups (4.2 vs 4.1 episodes per week).

The frequency of vomiting episodes (Table 3) also increased significantly in both groups after hospital discharge (baseline). At postintervention, it was 46% lower in the intervention than in the TAU group (4.3 vs 7.9 episodes per week); this difference was statistically significant. At follow-up, the frequency of vomiting episodes in the intervention group was 41% lower than in the TAU group (5.1 vs 8.7 episodes per week), but the difference was no longer significant. The frequency of episodes of all compensatory behaviors (Table 3) increased significantly in both groups after hospital discharge (baseline). Although at postintervention the frequency of episodes of all compensatory behaviors in the intervention group was 30% lower than in the TAU group (6.8 vs 9.8 episodes per week), the difference was not statistically significant. At follow-up, the frequency of episodes of all compensatory behaviors in the intervention group was 35% lower than in the TAU group (7.2 vs 11.1 episodes per week), but the difference was not statistically significant.

At postintervention, 37 (43.5%) women in the IN@ group and 30 (36.5%) in the TAU group who provided data (CCs set) did not meet diagnostic criteria for any eating disorder anymore; the difference was not significant (*P*=.43); whereas, 25 women (29.4%) in the IN@ group and 37 women (45.1%) in the TAU group met criteria for full syndrome BN, 19 (22.3%) in the IN@ group and 9 (11.0%) in the TAU group met criteria for atypical BN, 1 woman in the IN@ group had crossed over to binge eating disorder and 1 woman in the TAU group had crossed over to binge-eating/purging type AN.

At follow-up, 44 (55.6%) women in the IN@ group and 39 (45.8%) in the TAU group who provided data did not meet diagnostic criteria for any eating disorder anymore; the difference was not significant (*P*=.27). However, 19 women (24.1%) in the IN@ group and 28 women (32.9%) in the TAU group met criteria for full syndrome BN; 14 women (17.7%) in the IN@ group and 17 (20.0%) in the TAU group met criteria for atypical BN; 2 women in the IN@ group had crossed over to binge eating disorder and 1 woman in the TAU group had crossed over to binge-eating/purging type AN.

### Moderator Analyses

The following variables were identified as candidate moderators for abstinence at postintervention and follow up: number of diagnostic criteria for BN still present at baseline (hospital discharge), BMI at baseline, abstinence from binge eating and compensatory behaviors, frequency of episodes of binge eating, vomiting and all compensatory behaviors in the 2 weeks preceding baseline, and SIAB subscale scores at baseline.

Mixed model analyses revealed a significant group × variable interaction effect only for abstinence from binge eating both at postintervention and follow-up (Table 5).

Within the subgroup of patients who had failed to achieve abstinence at baseline, women in the intervention group had 4.93 (95% CI 1.90-12.80) times greater odds of abstinence at postintervention and follow-up time points, on average (*P*=.001).

### Adverse Events

At the end of the intervention period, 13 women (14.8%) in the IN@ group and 13 women (16.5%) in the TAU group reported clinically relevant suicidal thoughts, with no difference between the two groups (Χ^2^_4_=3.8; *P*=.44). One of these women from the TAU group reported suicidal thoughts for the first time at the end of the intervention period; all other women had also reported suicidal thoughts at prebaseline or baseline. Four women (two from each group) reported suicide attempts during the intervention period. Three of those women had comorbid major depression.

**Table 5:** Moderation of abstinence from binge eating and compensatory behaviors at postintervention and follow-up: final estimation of fixed effects (Population-average model with robust standard errors).

Fixed effect^a^		Coefficient		Standard error		Odds ratio (95% CI)		*P* value	
Intercept		0.20		0.126		1.22 (0.948-1.568)		.12	
**Postintervention slope**									
	Intercept		−2.43		0.329		0.09 (0.046-0.168)		<.001
	Main effect: IN@ intervention		1.24		0.441		3.46 (1.451-8.233)		.005
	Main effect: baseline abstinence^b^		2.00		0.419		7.38 (3.230-16.858)		<.001
	Interaction effect: intervention X baseline abstinence^b^		−1.71		0.595		0.18 (0.056-0.585)		.004
**Follow-up slope**									
	Intercept		−2.70		0.313		0.07 (0.036-0.124)		<.001
	Main effect: IN@ intervention effect		1.61		0.4318		5.02 (2.146-11.742)		<.001
	Main effect: baseline abstinence^b^		2.21		0.409		9.08 (4.055-20.349)		<.001
	Interaction effect: intervention X baseline abstinence^b^		−1.82		0.595		0.16 (0.050-0.523)		.002

^a^Abstinence was coded as 1, nonabstinence as 0 in the model.

^b^Abstinence from binge eating and compensatory behaviors at baseline (hospital discharge).

**Table 6:** Utilization of treatment as usual (TAU) by group (two women in each group did not provide data on treatment utilization at follow-up).

Utilization of TAU		IN@^a^ N_post_=85 N_follow-up_=77	TAU^b^ N_post_=82 N_follow-up_=83
**Any psychotherapeutic or psychiatric outpatient treatment between baseline and postintervention, n (%)**		70 (82.3%)	67 (81.7%)
	No. of sessions, mean (SD)	21.7 (23.86)	22.9 (23.91)
**Any psychotherapeutic or psychiatric outpatient treatment between postintervention and follow-up, n (%)**		55 (71.4%)	61 (73.5%)
	No. of sessions, mean (SD)	23.2 (49.71)	25.4 (31.16)
Any psychotherapeutic or psychiatric inpatient treatment between baseline and postintervention, n (%)^c^		6 (7.1%)	2 (2.4%)
Any psychotherapeutic or psychiatric inpatient treatment between postintervention and follow-up, n (%)^c^		9 (11.6%)	7 (8.4%)

^a^IN@: Web-based aftercare intervention.

^b^TAU: Treatment as usual.

^c^Excludes short interventions for suicidal tendencies or substance abuse.

### Treatment Seeking

A high proportion of patients of both groups utilized psychotherapeutic or psychiatric outpatient treatment between baseline and postintervention as well as postintervention and 9-month follow-up (Table 6). Rates were almost identical in both groups for both time periods as were number of sessions (IN@: 44.9; TAU: 48.3). A small proportion of patients also utilized further inpatient treatment between hospital discharge and follow-up. Women of the intervention group who did not utilize any further treatment during the intervention period were more likely to terminate the IN@ intervention prematurely (7 out of 14 women [50.0%] opened less than 25% of program pages) than women who received additional CBT (7 out of 30 women [23.3%] opened less than 25% of program pages) or psychodynamic therapy (10 out of 26 women [38.5%] opened less than 25% of program pages). However, the type (CBT vs psychodynamic) or amount of TAU treatment was not significantly related to any of the reported outcomes.

## Discussion

### Principal Findings

This is one of the first studies to evaluate the efficacy of a program targeting maintenance or improvement of treatment gains achieved during inpatient treatment in patients with severe BN. On the basis of ITT analysis at T2, we found that individuals receiving IN@ did not differ significantly from individuals receiving TAU in rates of abstinence from binge eating and any compensatory behaviors (21.4% vs 18.9%) and these rates remained stable at follow-up. They were higher in the CCs (and PP) sample but also not significantly different between groups. Although the intervention resulted in a 28% lower frequency of binge eating episodes at posttreatment compared with the TAU group, this difference was not significant. However, at postintervention, the IN@ group achieved a 46% lower frequency of vomiting episodes and this difference was significant. At follow up, this frequency was still 41% lower than in the TAU group but the difference was no longer significant. Moderator analyses revealed that both at post-intervention and follow-up, women of the intervention group who still reported binge eating and compensatory behaviors after the inpatient treatment had higher odds of achieving abstinence at post-intervention and follow-up compared with women of the TAU group who still reported binge eating and compensatory behaviors after the inpatient treatment.

### Comparison With Prior Work

The results of this study have to be discussed in the light of several important points: (1) general lack of comparison of maintenance studies for patients with BN, (2) illness severity, (3) additional treatment utilization and health care system in general, and (4) intervention characteristics.

For the primary outcome, abstinence of binge eating and any compensatory behaviors, only one other study for patients with BN has demonstrated that an aftercare intervention yields a significant reduction in abstinence while one other study did not find any differences on a number of self-report measures between the groups. In the first study, a 16-week, SMS for patients with BN after hospital discharge was investigated [36]. This relatively short intervention yielded significantly larger abstinence and partial remission rates at 8 months after hospital discharge than our intervention but the SMS-study differs in terms of duration of intervention and follow up, diagnoses (more EDNOS cases), and symptom frequency from ours. Additionally, treatment utilization in this study was 8-9 sessions over the 8 months of the intervention and follow-up compared with 27-29 sessions over 9 months of the intervention in our study. Because this study [36] was carried out as part of the same health care system as our own study, patients were probably not as severely ill as patients included in our study. In the second study [39] published after the starting of our study, both the Web-based support group and the waiting list group improved during the 4-month intervention with no significant differences between groups. Neither abstinence rates nor follow-up data were reported.

A second important point for the interpretation of the results of this study regards illness severity of the included patient group, indicated by symptom frequency, illness duration, and previous in- and outpatient treatment utilization. Patients in our study reported an average frequency of almost 15 binge eating episodes and over 18 vomiting episodes per week during the preceding 3 months before hospital admission. Although patients treated as inpatients may have higher frequencies of binge eating and purging episodes in general, these numbers clearly exceed frequencies reported in both earlier and more recent intervention trials for BN (eg, [50-52]). Our sample may therefore represent a more severely ill sample of BN patients which may limit the generalizability of the results.

The third crucial issue to be considered in the interpretation of the results is the amount of additional treatment utilization. Our study was originally planned to compare the efficacy of an aftercare intervention with TAU. However, at the time of planning the study, it was unclear what the “usual” amount of further treatment utilized by patients after hospital discharge would be, that is, whether and to what degree patients are recommended to take up further treatment following their inpatient stay and to what degree they would follow this recommendation. In this study, treatment utilization was surprisingly high: 82% of both groups engaged in additional treatment with 2-3 sessions per month during the 9-month intervention period and even higher numbers over the 9-month follow-up period. For patients, as severely ill as in this study, a Web-based aftercare intervention may have been less acceptable as stand-alone treatment compared with face-to-face treatment.

Finally, treatment utilization in this study may also be reflective of the specific health care context in which the study was conducted, that is, access to outpatient psychotherapeutic care and coverage of up to 80 sessions of outpatient psychotherapeutic treatment by health insurances in Germany. However, waiting times can be 6-12 months depending on regional differences. Because patients originated from different parts in Germany, the amount of further treatment utilization is even more surprising.

The characteristics of the intervention itself may also need to be discussed for the interpretation of the results. Adherence is a problem for Web-based interventions in general, also labeled as “law of attrition” [53]. Compared with targeted preventive interventions for eating disorders in student populations, in which adherence, defined as the number of opened pages and/or completed sessions, ranges between 50% and 80% [40], adherence to the IN@ intervention was lower. Participants opened on average 36% of the program pages; 15.1% of patients never logged on to the program after randomization and only 19.1% completed all 11 sessions of the program. However, the duration of preventive interventions is usually 8-10 weeks compared with 9 months in our study. For the included patients, the frequency of provided Web-based sessions especially during the first months may have been too low and the duration of the intervention and of individual sessions may have been too long. Future maintenance interventions may benefit from an overall shorter intervention duration and shorter, more frequent sessions which may also improve adherence to the intervention.

### Clinical and Service-Related Implications

Although the intervention did not have a significant effect on abstinence rates at T2, the results of secondary outcomes, specifically vomiting rates, and moderator analyses may have important clinical implications: In the IN@ group, rates of vomiting episodes were almost half the rates of patients in the TAU group. From a clinical perspective, reducing vomiting episodes is often more difficult for patients than reducing binge eating, and while patients may have given up or reduced the frequency and quality of binge eating episodes considerably, vomiting is often the more persistent symptom. Because vomiting is also specifically associated with severe medical complications, its reduction during treatment is of essential clinical importance [54]. However, despite the marked reductions in vomiting rates during the intervention and follow-up, average rates were still above the diagnostically relevant threshold.

The intervention also proved to be specifically beneficial for patients who had not achieved abstinence at the time of hospital discharge. Thus, for a subgroup of potentially even more severely ill patients, participating in the aftercare intervention turned out to be specifically advantageous. In our study, only 54.9% of patients achieved abstinence of all BN symptoms during inpatient treatment in the past 2 weeks before baseline. Accordingly, a considerable proportion of patients were in need of further or more specific support to achieve full remission. Generally, the lack of studies examining short- and long-term effects of inpatient or residential treatments limits the comparability of these outcomes [55]. However, studies addressing long-term course and outcome of BN often demonstrate that relapses after the end of treatment are common and that patient status at posttreatment does not necessarily predict recovery status at follow-up [56]. The fact that patients in this study did not maintain improvements achieved during inpatient treatment and that a large proportion of patients did not improve markedly despite further treatment utilization and participation in IN@ may also demonstrate the need for a stepped—and more consistent and specialized—aftercare treatment provision. TAU after inpatient treatment is obviously provided irrespective of previous treatment gains or further needs in specific symptom domains. Although for some patients the aftercare intervention may have been sufficient to maintain their treatment gains, others may have needed even more intensive outpatient face-to-face treatment. To answer the question of who might need which amount of further Web-based or face-to-face treatment would require a change in current provision of health care delivery based on evidence rather than nonevidence based exploitation of existing health care structures.

### Strengths and Limitations

Our study includes one of the largest sample sizes of patients of intervention trials for patients with BN, which clearly is a strength of the study. Follow-up duration is relatively long, BN symptoms and diagnoses were ascertained by a well-validated clinical interview, and the number of adverse events was not higher in IN@ compared with TAU. In a stepped-care model, the intervention could represent an economic alternative to face-to-face treatment if provided in a shortened version as first step and full alternative to TAU.

Limitations of the study are moderately higher dropout rates compared with face-to-face intervention trials and low adherence to the Web-based intervention. Future research efforts should therefore also be directed at adapting the intervention to increase adherence to the program and reduce dropouts.

### Conclusions

Taken together, data from this study suggest moderate effects of IN@ for patients with severe and chronic BN. With regard to abstinence rates, IN@ did not show a marked superiority over TAU. However, the intervention had encouraging effects on vomiting rates and might also be particularly beneficial for women who have not achieved abstinence during previous inpatient treatment when compared with TAU.

## Appendix

Multimedia Appendix 1 IN@ screenshot 1.

Multimedia Appendix 2 IN@ screenshot 2.

Multimedia Appendix 3 CONSORT-EHEALTH checklist (v1.6.1).

## Abbreviations

AN: anorexia nervosa
BN: Bulimia nervosa
BMI: body mass index
CBT: cognitive behavioral therapy
DSM-IV: Diagnostic and Statistical Manual of Mental Disorders
RP: relapse prevention
SMS: short message service
TAU: treatment as usual

## References

[1] Hudson JI, Hiripi E, Pope HG, Kessler RC (2007). The prevalence and correlates of eating disorders in the National comorbidity survey replication. Biol Psychiatry.

[2] Swanson SA, Crow SJ, Le Grange D, Swendsen J, Merikangas KR (2011). Prevalence and correlates of eating disorders in adolescents. Results from the national comorbidity survey replication adolescent supplement. Arch Gen Psychiatry.

[3] Steinhausen H, Weber S (2009). The outcome of bulimia nervosa: findings from one-quarter century of research. Am J Psychiatry.

[4] Castellini G, Lo SC, Mannucci E, Ravaldi C, Rotella CM, Faravelli C, Ricca V (2011). Diagnostic crossover and outcome predictors in eating disorders according to DSM-IV and DSM-V proposed criteria: a 6-year follow-up study. Psychosom Med.

[5] Bulik CM, Sullivan PF, Joyce PR, Carter FA, McIntosh VV (1998). Predictors of 1-year treatment outcome in bulimia nervosa. Compr Psychiatry.

[6] Turnbull SJ, Schmidt U, Troop NA, Tiller J, Todd G, Treasure JL (1997). Predictors of outcome for two treatments for bulimia nervosa: short and long-term. Int J Eat Disord.

[7] Fahy TA, Russell GF (1993). Outcome and prognostic variables in bulimia nervosa. Int J Eat Disord.

[8] Olmsted MP, Kaplan AS, Rockert W (1994). Rate and prediction of relapse in bulimia nervosa. Am J Psychiatry.

[9] Helverskov JL, Clausen L, Mors O, Frydenberg M, Thomsen PH, Rokkedal K (2010). Trans-diagnostic outcome of eating disorders: a 30-month follow-up study of 629 patients. Eur Eat Disord Rev.

[10] Marrone S, Mitchell JE, Crosby R, Wonderlich S, Jollie-Trottier T (2009). Predictors of response to cognitive behavioral treatment for bulimia nervosa delivered via telemedicine versus face-to-face. Int J Eat Disord.

[11] Wilson GT, Loeb KL, Walsh BT, Labouvie E, Petkova E, Liu X, Waternaux C (1999). Psychological versus pharmacological treatments of bulimia nervosa: predictors and processes of change. J Consult Clin Psychol.

[12] National Institute for Health and Care Excellence (2004). Eating disorders: Core interventions in the treatment and management of anorexia nervosa, bulimia nervosa and related eating disorders.

[13] Herpertz S, Herpertz-Dahlmann B, Fichter M, Tuschen-Caffier B, Zeeck A (2011). S3-Leitlinie Diagnostik und Behandlung der Essstörungen.

[14] Fairburn C, Marcus M, Wilson G (1993). Cognitive-behavioral therapy for binge eating and bulimia nervosa: a comprehensive treatment manual. Binge eating: Nature, assessment, and treatment.

[15] Hay P (2013). A systematic review of evidence for psychological treatments in eating disorders: 2005-2012. Int J Eat Disord.

[16] Hay PP, Bacaltchuk J, Stefano S, Kashyap P (2009). Psychological treatments for bulimia nervosa and binging. Cochrane Database Syst Rev.

[17] Olmsted MP, Kaplan AS, Rockert W (2005). Defining remission and relapse in bulimia nervosa. Int J Eat Disord.

[18] Richard M, Bauer S, Kordy H (2005). Relapse in anorexia and bulimia nervosa—a 2.5-year follow-up study. Eur Eat Disorders Rev.

[19] Fichter MM, Quadflieg N, Hedlund S (2008). Long-term course of binge eating disorder and bulimia nervosa: relevance for nosology and diagnostic criteria. Int J Eat Disord.

[20] Mitchell JE, Agras WS, Wilson GT, Halmi K, Kraemer H, Crow S (2004). A trial of a relapse prevention strategy in women with bulimia nervosa who respond to cognitive-behavior therapy. Int J Eat Disord.

[21] Wonderlich S, Mitchell JE, Crosby RD, Myers TC, Kadlec K, Lahaise K, Swan-Kremeier L, Dokken J, Lange M, Dinkel J, Jorgensen M, Schander L (2012). Minimizing and treating chronicity in the eating disorders: a clinical overview. Int J Eat Disord.

[22] Ebert DD, Gollwitzer M, Riper H, Cuijpers P, Baumeister H, Berking M (2013). For whom does it work? moderators of outcome on the effect of a transdiagnostic internet-based maintenance treatment after inpatient psychotherapy: randomized controlled trial. J Med Internet Res.

[23] Ebert D, Tarnowski T, Gollwitzer M, Sieland B, Berking M (2013). A transdiagnostic internet-based maintenance treatment enhances the stability of outcome after inpatient cognitive behavioral therapy: a randomized controlled trial. Psychother Psychosom.

[24] Golkaramnay V, Bauer S, Haug S, Wolf M, Kordy H (2007). The exploration of the effectiveness of group therapy through an internet chat as aftercare: a controlled naturalistic study. Psychother Psychosom.

[25] Golkaramnay V, Wangemann T, Dogs J, Dogs P, Kordy H (2003). [New bridges for gaps in psychotherapeutic service provision by the internet: hopes, challenges and a solution]. Psychother Psychosom Med Psychol.

[26] Barnes C, Harvey R, Mitchell P, Smith M, Wilhelm K (2007). Evaluation of an online relapse prevention program for bipolar disorder - an overview of the aims and methodology of a randomized controlled trial. Disease Management & Health Outcomes.

[27] Moessner M, Aufdermauer N, Baier C, Göbel H, Kuhnt O, Neubauer E, Poesthorst H, Kordy H (2014). [Efficacy of an internet-delivered aftercare program for patients with chronic back pain]. Psychother Psychosom Med Psychol.

[28] Christensen H, Hickie IB (2010). E-mental health: a new era in delivery of mental health services. Med J Aust.

[29] Portnoy DB, Scott-Sheldon LAJ, Johnson BT, Carey MP (2008). Computer-delivered interventions for health promotion and behavioral risk reduction: a meta-analysis of 75 randomized controlled trials, 1988-2007. Prev Med.

[30] Beintner I, Jacobi C, Taylor CB (2012). Effects of an Internet-based prevention programme for eating disorders in the USA and Germany--a meta-analytic review. Eur Eat Disord Rev.

[31] Stice E, Shaw H, Marti CN (2007). A meta-analytic review of eating disorder prevention programs: encouraging findings. Annu Rev Clin Psychol.

[32] Jacobi C, Völker U, Trockel MT, Taylor CB (2012). Effects of an internet-based intervention for subthreshold eating disorders: a randomized controlled trial. Behav Res Ther.

[33] Taylor CB, Bryson S, Luce KH, Cunning D, Doyle AC, Abascal LB, Rockwell R, Dev P, Winzelberg AJ, Wilfley DE (2006). Prevention of eating disorders in at-risk college-age women. Arch Gen Psychiatry.

[34] Stice E, Shaw H, Becker CB, Rohde P (2008). Dissonance-based interventions for the prevention of eating disorders: using persuasion principles to promote health. Prev Sci.

[35] Ohlmer R, Jacobi C, Taylor CB (2013). Preventing symptom progression in women at risk for AN: results of a pilot study. Eur Eat Disord Rev.

[36] Bauer S, Okon E, Meermann R, Kordy H (2012). Technology-enhanced maintenance of treatment gains in eating disorders: efficacy of an intervention delivered via text messaging. J Consult Clin Psychol.

[37] Fichter MM, Quadflieg N, Nisslmüller K, Lindner S, Osen B, Huber T, Wünsch-Leiteritz W (2012). Does internet-based prevention reduce the risk of relapse for anorexia nervosa?. Behav Res Ther.

[38] Fichter MM, Quadflieg N, Lindner S (2013). Internet-based relapse prevention for anorexia nervosa: nine- month follow-up. J Eat Disord.

[39] Gulec H, Moessner M, Túry F, Fiedler P, Mezei A, Bauer S (2014). A randomized controlled trial of an internet-based posttreatment care for patients with eating disorders. Telemed J E Health.

[40] Beintner I, Jacobi C, Taylor CB (2014). Participant adherence to the internet-based prevention program student bodies? for eating disorders - a review. Internet Interv.

[41] Beintner I, Jacobi C (2011). Internetgestützte Nachsorge bei Bulimia Nervosa. Psychotherapeut.

[42] Fichter MM, Herpertz S, Quadflieg N, Herpertz-Dahlmann B (1998). Structured interview for anorexic and bulimic disorders for DSM-IV and ICD-10: updated (third) revision. Int J Eat Disord.

[43] Wittchen H, Zaudig M, Fydrich T (1997). SKID: Strukturiertes Klinisches Interview für DSM-IV: Achse I und II.

[44] Raudenbush S, Bryk A (2002). Hierarchical Linear Models: Applications and Data Analysis Methods.

[45] Chakraborty H, Gu H (2009). RTI.

[46] Peters SAE, Bots ML, den Ruitjer HM, Palmer MK, Grobbee DE, Crouse JR, O'Leary DH, Evans GW, Raichlen JS, Moons KGM, Koffijberg H (2012). Multiple imputation of missing repeated outcome measurements did not add to linear mixed-effects models. J Clin Epidemiol.

[47] Twisk J, de Boer M, de Vente M, Heymans M (2013). Multiple imputation of missing values was not necessary before performing a longitudinal mixed-model analysis. J Clin Epidemiol.

[48] Kraemer HC, Stice E, Kazdin A, Offord D, Kupfer D (2001). How do risk factors work together? mediators, moderators, and independent, overlapping, and proxy risk factors. Am J Psychiatry.

[49] Kraemer HC, Wilson GT, Fairburn CG, Agras WS (2002). Mediators and moderators of treatment effects in randomized clinical trials. Arch Gen Psychiatry.

[50] Agras WS, Walsh T, Fairburn CG, Wilson GT, Kraemer HC (2000). A multicenter comparison of cognitive-behavioral therapy and interpersonal psychotherapy for bulimia nervosa. Arch Gen Psychiatry.

[51] Poulsen S, Lunn S, Daniel SI, Folke S, Mathiesen BB, Katznelson H, Fairburn CG (2014). A randomized controlled trial of psychoanalytic psychotherapy or cognitive-behavioral therapy for bulimia nervosa. Am J Psychiatry.

[52] Mitchell JE, Agras S, Crow S, Halmi K, Fairburn CG, Bryson S, Kraemer H (2011). Stepped care and cognitive-behavioural therapy for bulimia nervosa: randomised trial. Br J Psychiatry.

[53] Eysenbach G (2005). The law of attrition. J Med Internet Res.

[54] Mehler PS, Rylander M (2015). Bulimia Nervosa - medical complications. J Eat Disord.

[55] Friedman K, Ramirez AL, Murray SB, Anderson LK, Cusack A, Boutelle KN, Kaye WH (2016). A narrative review of outcome studies for residential and partial hospital-based treatment of eating disorders. Eur Eat Disord Rev.

[56] Lock J, Agras WS, Le Grange D, Couturier J, Safer D, Bryson SW (2013). Do end of treatment assessments predict outcome at follow-up in eating disorders?. Int J Eat Disord.

