# Supplementary figures and images for: Web-Based Aftercare for Women With Bulimia Nervosa Following Inpatient Treatment: Randomized Controlled Efficacy Trial
Source: J Med Internet Res. 2017 Sep 22;19(9):e321. doi: 10.2196/jmir.7668 (PMC5630693; doi:10.2196/jmir.7668)

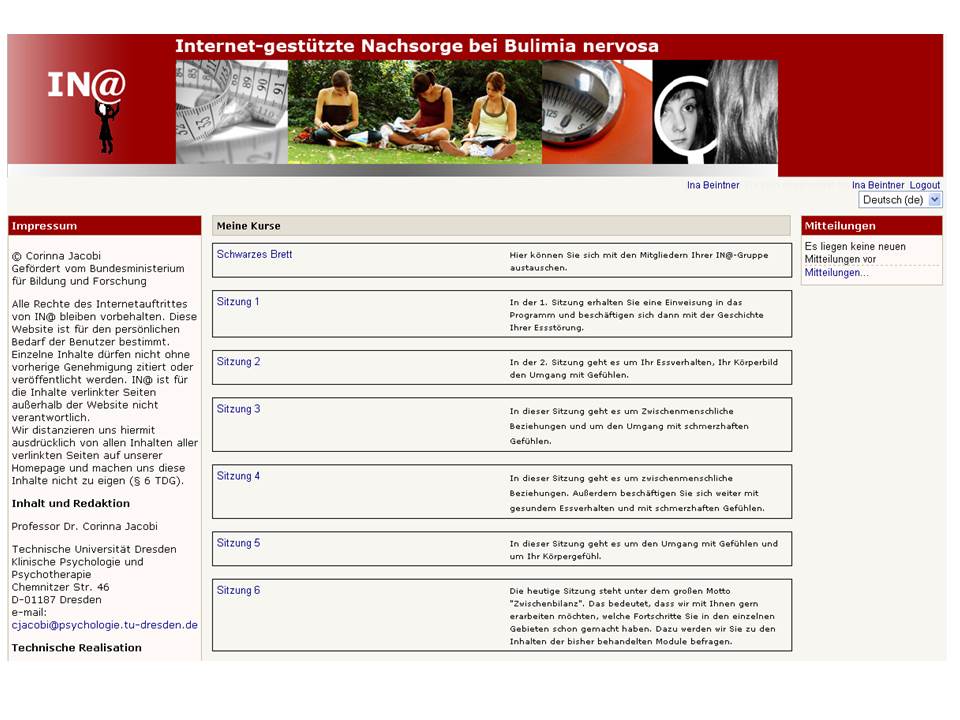

Supplement: Multimedia Appendix 1 [file jmir_v19i9e321_app1.JPG]

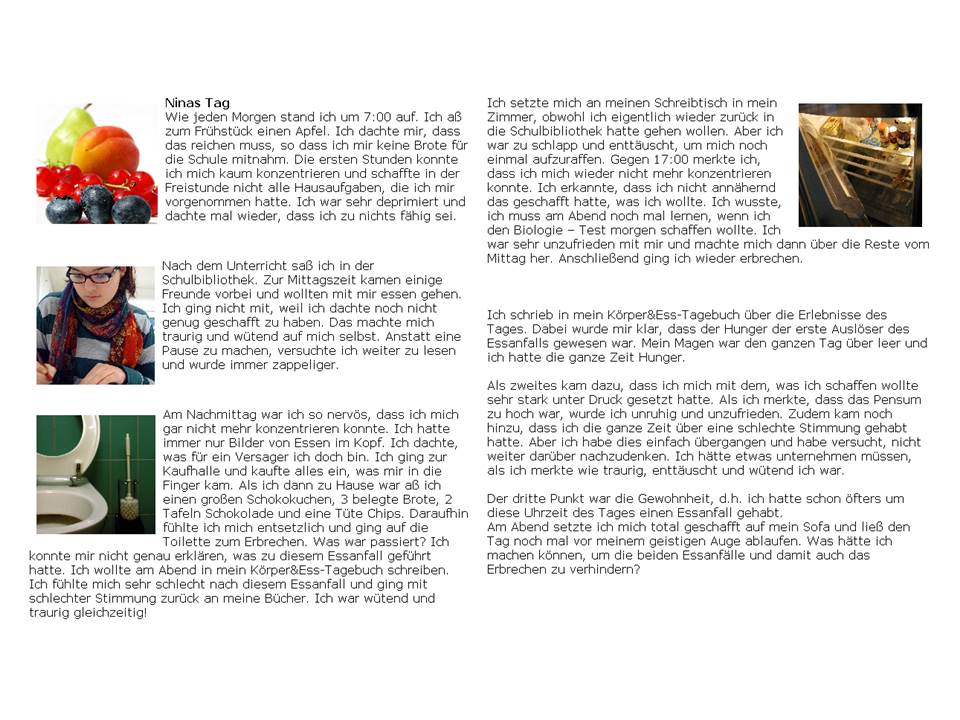

Supplement: Multimedia Appendix 2 [file jmir_v19i9e321_app2.JPG]
